# Patient Factors Associated With the Use of Online Portal Health Information in the Postpandemic Era: Cross-Sectional Analysis of a National Survey

**DOI:** 10.2196/60472

**Published:** 2025-04-03

**Authors:** Ishana Maini, Kevin Gilotra, Gelareh Sadigh

**Affiliations:** 1 Chicago College of Osteopathic Medicine Midwestern University Downers Grove, IL United States; 2 Renaissance School of Medicine Stony Brook University Stony Brook, NY United States; 3 Department of Radiological Sciences University of California Irvine Orange, CA United States

**Keywords:** patient portal, utilization, health disparity, post-pandemic, health information, prevalence, portal use, survey, health care provider, Americans, US

## Abstract

**Background:**

Patients’ electronic access to their health information can improve long-term health outcomes. Few studies have evaluated barriers that may limit access to portal health information before the COVID-19 pandemic such as preference for in-person visits, lack of perceived need to use a patient portal system, and lack of comfort or experience with computers. With the increased use of telehealth during the pandemic, patients’ comfort with portal applications and digital health literacy has improved.

**Objective:**

The purpose of this study was to assess the prevalence of portal use and factors associated with patients’ portal access after the COVID-19 pandemic.

**Methods:**

This study used data from the 2022 National Cancer Institute’s Health Information National Trends Survey (HINTS 6). Adult patients (aged ≥18 years) who responded to the survey question about patient portal access were included. A multivariate logistic regression analysis was performed to determine characteristics associated with portal access.

**Results:**

A total number of 5958 patients were included (weighted n=245,721,106), with a mean age of 48.2 (20.1) years and were mostly female (119,538,392/236,138,857, 50.6%) and white (167,163,482/227,232,636, 73.6%). Overall, 61.3% (150,722,178/245,721,106) of all respondents reported accessing portals over the last 12 months and 43.7% (82,620,907/188,860,031) used multiple portals. Most participants (135,011,661/150,104,795, 89.9%) reported using portals to access test results, followed by viewing clinical notes (104,541,142/149,867,276, 69.8%) downloading personal health information (47,801,548/150,017,130, 31.9%). The likelihood of portal use significantly increased by 24.9% points (95% CI 19.4-30.5) when patients were offered access to portals by health care providers or insurers compared with those not offered access or did not know if they were offered access. The likelihood of portal use also increased by 19.5% points (95% CI 15.1-23.9) among patients with a health care provider encouraging them to access portals, compared to patients who did not receive encouragement to do so. Having a college education versus education below college level and living in metropolitan areas versus nonmetropolitan regions increased the probability of portal use by 6.9% points (95% CI 3.1-10.8) and 6.9% points (95% CI 1.3-12.6), respectively. Of note, males (compared with females) and those of Hispanic background (compared with non-Hispanic individuals) were less likely to be offered portal access by 10.8% points (95% CI 6.3-15.2) and 6.9% points (95% CI 1.7-12.1), respectively.

**Conclusions:**

This study demonstrates that most Americans use patient portals, though certain patient populations such as those with less than college degree education or living in nonmetropolitan areas continue to face greater difficulty accessing them. Interventions targeted at equality in offering access to patient portals and encouraging patients to use them could advance equitable and widespread access to patient portals.

## Introduction

Electronic health tools are becoming increasingly available as they help facilitate patient independence within the health care system [1,2]. Patient portals are a unique tool allowing patients to access, manage, and share their medical records outside clinical care settings [2]. Potential uses of patient portals include viewing appointment summaries, test results, and medication lists. By empowering patients to take ownership over their health while improving patient-physician communication in the outpatient setting, patient portals offer many tangible benefits that may improve long-term health outcomes [2]. Furthermore, accessing patient records supplements in-person communication with providers and reduces patient reliance on in-person visits for accessing pertinent health information [3].

Despite the reported benefits of patient portal usage, as of 2019, only 37% of Americans were accessing patient portals nationwide [4], which increased to 68% in 2022 among patients who had health care visits in the last 12 months [5]. Before the COVID-19 pandemic, among individuals who did not use a patient portal, the most common reasons were a preference for in-person visits and a lack of perceived need to use a patient portal system. Other barriers included a lack of comfort or experience with computers, having no internet access, and privacy concerns [6,7]. With the increased use of telehealth during the pandemic, patients’ comfort with portal applications and digital health literacy has improved [8], and therefore, it is important to re-evaluate patients’ factors associated with more portal use. Studies published after the pandemic have predominantly focused on racial and ethnic disparities in portal use [5,9]. However, disparities across other patient factors are less studied.

The purpose of this study was to assess patient factors associated with patient access to online health portals after the COVID-19 pandemic. Understanding these factors can allow health care providers and organizations to better engage their patients in the use of electronic health care tools such as patient portals.

## Methods

This publicly available survey study did not use any private identifiable information and thus did not constitute human subject research requiring institutional review board oversight, falling under exempt category 2 of our institutional Human Research Protections guidelines [10]. An administrative exempt self-determination was filed with our institutional review board (IRB#6379).

### Study Design and Data Source

We performed a cross-sectional analysis of data from the Health Information National Trends Survey 6 (HINTS 6) [11]. HINTS 6 is a national survey conducted by the National Cancer Institute in 2022. The target population was civilian, noninstitutionalized adults over the aged of 18 years residing in the United States. The intention of the survey was to understand the American public’s use of cancer-related information. Participants were recruited based on a random sample of addresses stratified by rural or urban designation and stratified by minority concentration in the region. One adult was selected from each household and offered the opportunity to respond to the survey in either paper or web-based formats.

### Study Population

HINTS 6 recruited a nationally representative sample of 6252 individuals with a 28.1% overall response rate. For this study, participants who had missing responses to the question inquiring about access to patient portals (294/6252) were excluded, and therefore, 5958 individuals were included in this study sample.

### Measures

The primary outcome was access to patient portals, determined based on the survey question, “How many times did you access your online medical record or patient portal in the last 12 months?” Those who indicated zero instances of portal access were labeled as nonusers, while respondents who selected any nonzero number were labeled as users of patient portals.

Secondary outcomes included reported use of multiple online portals, use of a portal organizer app, functions used within portals, health care providers or insurers offering access to patient portals, and health care providers encouraging access to portals. The use of multiple online portals was assessed through the survey question, “Do you have one, or more than one patient portal or online medical record?” with responses categorized as “one” versus “more than one.” Respondents indicating the use of “more than one” were asked about portal organizer app usage through a yes or no question, “Have you ever used an app like ‘Apple Health Records’ or ‘CommonHealth’ to combine your medical information from different patient portals or online medical records into one place?” In addition, functions used within patient portals were also assessed through a single question, “In the past 12 months, have you used your online medical record or patient portal to…” with answer options: (1) lookup test results, (2) download personal health information, (3) electronically send health information to a third party, and (4) view clinical notes.

Patients were also asked, “Have you ever been offered online access to your medical records (for example, a patient portal) by your health care provider or health insurer?” The response options allowed patients to select either “yes,” “no,” or “don’t know,” separately for health care provider or insurer. Encouragement of portal use by health care providers was assessed using responses to the yes or no question “Have any of your health care providers, including doctors, nurses, or office staff ever encouraged you to use an online medical record or patient portal?”

The independent variables included patients’ sociodemographics. These included age, sex, race, ethnicity, marital status, education, household income, and health insurance. Rural or urban living was assessed using the 2013 Rural-Urban Continuum Codes, designed to delineate metropolitan and nonmetropolitan areas based on population size. Patients’ satisfaction with internet connection at home, and report of at least one health-related social need in the last 12 months (food insecurity, housing instability, and transportation access problem) as a proxy for individual socioeconomic status was collected as independent variables as may impact patients’ utilization of portal [12]. Patient well-being was assessed by frequency of provider visits in the previous 12 months, overall quality of health care received in the past 12 months, general health as a proxy for quality of life, and existing medical conditions (eg, cancer, diabetes, etc). Health literacy was determined based on a single question about participant confidence in filling out medical forms, which has been validated as useful in identifying inadequate health literacy [13], and has been shown to be associated with portal use [8].

### Analyses

All data were analyzed using survey weights based on population estimates to account for nonresponse and coverage error, making the results more generalizable to the population, based on HINTS protocols. As is best practice with the HINTS data, jackknife replicate weights were used to provide bias‐corrected variance estimates [11].

Descriptive statistics were used to summarize the sample’s sociodemographic and clinical characteristics as well as patient portal access in the last 12 months, functions used within the portal, and health care or insurer offering or encouraging access to portals. A weighted percentage for descriptive statistics is reported.

Weighted univariable logistic regression models using average marginal effect were used to determine which sociodemographic and clinical characteristics (independent variables) were associated with patient use or nonuse of patient portals (primary outcome) as well as offering access to patient portals by either health care provider or insurer (secondary outcome). Selected independent variables were those that have either been associated with portal use in previous literature (eg, health literacy [8], race [14], internet access or socioeconomic status [12]) or expected to be associated with portal use. A weighted multivariable logistic regression model was constructed. Independent variables included in the model were those that were statistically significant in the univariable model or have been shown to be associated with the outcome [8,14] or were expected to be associated with the outcome. Multicollinearity among variables was examined. All data analysis was conducted using Stata (StataCorp) or MP 17.0 and SPSS (version 29; IBM SPSS Statistics). Statistical significance was defined as a *P* value of <.05.

## Results

### Sample Characteristics

The sample population consisted of 5958 individuals (weighted n=245,721,106). A majority of participants were female (119,538,392/236,138,857, 50.6%) and White (167,163,482/227,232,636, 73.6%), with a mean age of 48.2 (20.1) years. A total of 33.1% (77,726,009/234,878,511) were college graduates or higher, and 63.1% (142,029,743/225,019,936) of the sample indicated an annual household income of greater than US $50,000. A large percentage of participants resided in metropolitan regions (215,234,746/245,721,106, 87.6%), compared with rural areas. The baseline characteristics of participants are shown in Table 1.

**Table 1:** Baseline characteristics of study participants (N=5958; n=unweighted sample size; %=weighted percentage).

Characteristics			Participants, n (%)
**Demographics**			
	**Age (years; missing: n=76), n (%)**		
		18-40	1396 (21.3)
		41-64	2393 (43.3)
		More than 65	2203 (35.4)
	**Sex (missing: n=255), n (%)**		
		Female	3442 (50.6)
		Male	2261 (49.4)
	**Race (missing: n=455), n (%)**		
		Asian	299 (5.9)
		Black	999 (12.6)
		Other	321 (7.9)
		White	3884 (73.6)
	**Ethnicity (missing: n=460), n (%)**		
		Not Hispanic or Latino	4526 (83.3)
		Hispanic or Latino	972 (16.7)
	**Marital status (missing: n=265), n (%)**		
		Married	2573 (51.0)
		Not married (other)	3120 (49.0)
	**Education (missing: n=251), n (%)**		
		College graduate or higher	2694 (33.1)
		Below college level	3013 (66.9)
	**Annual household income (US $; missing: n=552), n (%)**		
		<50,000	2331 (36.9)
		≥50,000	3075 (63.1)
	**Rural or urban communities (no data missing), n (%)**		
		Metro	5185 (87.6)
		Nonmetro	773 (12.4)
**Social determinants of health**			
	**Health insurance (missing: n=36), n (%)**		
		Insured	5429 (89.4)
		Uninsured	493 (10.6)
	**Report of at least one health-related social need in the last 12 months^a^ (missing: n=188), n (%)**		
		Never	5213 (75.6)
		Yes, sometimes, or often	582 (24.4)
	**Satisfaction with internet connection at home (missing: n=1030), n (%)**		
		Not satisfied	1645 (32.1)
		Satisfied	3283 (67.9)
**Health characteristics and health care provider interactions**			
	**Frequency of provider visits in the last 12 months (missing: n=53), n (%)**		
		Never	670 (13.9)
		At least once	5235 (86.1)
	**Quality of health care received in the past 12 months (missing: n=726), n (%)**		
		Poor or fair	450 (8.3)
		Good, very good, or excellent	4782 (91.7)
	**Existing medical conditions (missing: n=68), n (%)**		
		No medical conditions	1712 (36.3)
		At least one medical condition	4178 (63.7)
	**General health (missing: n=83), n (%)**		
		Poor or fair	1047 (16.8)
		Good, very good, or excellent	4828 (83.2)
	**Confident filing medical forms (missing: n=19), n (%)**		
		No	788 (14.4)
		Yes	5429 (85.6)

^a^Includes food insecurity, housing instability, and transportation access problem.

### Patient Portal Access

A total of 61.3% (150,722,178/245,721,106) of respondents accessed patient portals within the past year. Of those, 33.4% (50,353,899/150,722,178) and 31.5% (47,438,401/150,722,178) indicated they accessed their portals between 1 to 2 times and 3-5 times, respectively. Furthermore, 43.7% (82,620,907/188,860,031) of participants reported using multiple patient portals from different health care providers. The most prevalent reason for portal use was to view test results, with 89.9% (135,011,661/150,104,795) of participants indicating they use this function. Other reasons for use included viewing clinical notes (104,541,142/149,867,276, 69.8%), downloading personal health information (47,801,548/150,017,130, 31.9%), and sending health information to third parties (30,679,355/150,044,002, 20.4%). Only 5.4% used an app like Apple health records or common health to combine their medical information from different portals into 1 place. A total of 71.4% (175,013,396/ 244,878,859) and 68.4% (167,239,592/244,637,179) reported that their health care provider offered and encouraged them to access patient portals, respectively, while 38.9% (89,161,906/229,388,136) were offered access to portals by their insurers. Overall, 75% (180,528,121/240,774,212) reported being offered portal access either by health care provider or insurer (Table 2).

**Table 2:** Patient portal access among study participants (N=5958; n=unweighted sample size; %=weighted percentage).

Characteristics			Participants, n (%)
**Patient portal access in the last 12 months (no missing data), n (%)**			
	Never	2304 (38.7)	
	One time or more	3654 (61.3)	
**Using multiple online portals (missing: n=1428), n (%)**			
	Only one	2544 (56.3)	
	More than one	1986 (43.7)	
**Using portal organizer app (missing: n=3894), n (%)**			
	No	1870 (94.6)	
	Yes	104 (5.4)	
**Health care provider offering access to portals (missing: n=37), n (%)**			
	No or don’t know	1614 (28.5)	
	Yes	4307 (71.5)	
**Health care provider encouraging access to portals (missing: n=35), n (%)**			
	No	1913 (31.6)	
	Yes	4010 (68.4)	
**Insurer offering access to portals (missing: n=530), n (%)**			
	No or don’t know	3336 (61.1)	
	Yes	2092 (38.9)	

### Associations Between Sample Characteristics and Portal Use

Univariable and multivariable analyses of factors associated with portal use are shown in Table 3. In multivariable analysis, the likelihood of portal use was 24.9% points higher (95% CI 19.4-30.5) when patients were offered access to a portal through their health care providers or insurer. Similarly, portal usage was 19.5% points higher (95% CI 15.1-23.9) if a health care provider encouraged a patient to access portals. Finally, having a college education and living in urban areas were associated with 6.9% points (95% CI 3.1-10.8) and 6.9% points (95% CI 1.3-12.6), higher likelihood of portal use, respectively.

**Table 3:** Univariable and multivariable factors associated with portal use.

Characteristics				Univariable analysis average marginal effect (95% CI)		*P* value			Multivariable analysis average marginal effect (95% CI)		*P* value		
**Demographics**													
	**Age (years)**												
		18-40	Reference		—^a^			Reference		—			
		41-64	0.034 (–0.023 to 0.093)		.23			–0.003 (0.051 to 0.045)		.90			
		More than 65	–0.103 (–0.163 to –0.047)		.001			-0.032 (-0.086, 0.022)		.24			
	**Sex**												
		Female	Reference		—			Reference		—			
		Male	–0.123 (–0.165 to –0.083)		<.001			–0.039 (0.080 to 0.001)		.06			
	**Race**												
		Asian	0.006 (–0.139 to 0.152)		.93		0.045 (–0.057 to 0.147)						.38
		Black	–0.112 (–0.173 to –0.050)		<.001		–0.019 (–0.089 to 0.051)						.59
		Other	–0.079 (–0.196 to 0.038)		.18		0.055 (–0.023 to 0.133)						.16
		White	Reference		—		Reference						—
	**Ethnicity**												
		Not Hispanic or Latino	Reference		—			Reference		—			
		Hispanic or Latino	–0.151 (–0.214 to –0.088)		<.001			–0.027 (–0.092 to 0.038)		.41			
	**Marital status**												
		Not married (other)	Reference		—			Reference		—			
		Married	0.151 (0.102 to 0.200)		<.001			0.045 (–0.001 to 0.092)		.06			
	**Education**												
		Below college level	Reference		—			Reference		—			
		College graduate or higher	0.259 (0.219 to 0.298)		<.001			0.069 (0.031 to 0.108)		.001			
	**Annual household income (US $)**												
		<50,000	Reference		—			Reference		—			
		≥50,000	0.241 (0.198 to 0.284)		<.001			0.052 (–0.006 to 0.110)		.09			
	**Rural or urban communities**												
		Nonmetro	Reference		—			Reference		—			
		Metro	0.096 (0.042 to 0.151)		.001			0.069 (0.013 to 0.126)		.02			
**Social determinants of health**													
	**Health insurance**												
		Uninsured	Reference		—			Reference		—			
		Insured	0.245 (0.155 to 0.333)		<.001			–0.007 (–0.125 to 0.111)		.91			
	**Report of at least one health-related social need in the last 12 months** ^b^												
		Never	Reference		—			Reference		—			
		Yes, sometimes or often	–0.108 (–0.166 to –0.051)		<.001			0.010 (–0.048 to 0.069)		.73			
	**Satisfaction with internet connection at home**												
		Not satisfied	Reference		—			Reference		—			
		Satisfied	0.108 (0.058 to 0.157)		<.001			0.036 (–0.007 to 0.078)		.09			
**Health characteristics and health care provider interactions**													
	**Quality of care**												
		Not good	Reference		—			Reference		—			
		Good	0.143 (0.080 to 0.206)		<.001			0.013 (–0.064 to 0.091)		.73			
	**Medical conditions**												
		No medical conditions	Reference		—		—						—
		At least one medical condition	0.076 (0.036 to 0.116)		<.001		0.014 (–0.029 to 0.056)						.52
	**General health**												
		Poor or fair	Reference		—		Reference						—
		Good, very good, or excellent	0.091 (0.021 to 0.161)		.01		–0.020 (–0.083 to 0.042)						.51
	**Confident filing medical forms**												
		No	Reference		—			Reference		—			
		Yes	0.168 (0.108 to 0.228)		<.001			0.042 (–0.032 to 0.116)		.26			
	**Health care provider or insurer offering access to portals**												
		No or don’t know	Reference		—			Reference		—			
		Yes	0.486 (0.462 to 0.511)		<.001			0.249 (0.194 to 0.305)		<.001			
	**Health care provider encouraging access to portals**												
		No	Reference		—			Reference		—			
		Yes	0.438 (0.425 to 0.452)		<.001			0.195 (0.151 to 0.239)		<.001			

^a^Not applicable.

^b^Includes food insecurity, housing instability, and transportation access problem.

### Associations Between Sample Characteristics and Portal Access Offer

Univariable and multivariable analyses of factors associated with portal access offered by either health care provider or insurer are shown in Table 4. In multivariable analysis, the likelihood of being offered portal access was 8.6% points higher (95% CI 4.9-12.3) among married individuals, 7.1% points higher (95% CI 2.0-12.2) among college graduates, 7.2% points higher (95% CI 1.0-13.4) among those with annual household income of US $50,000 or more, 10.6% points higher (95% CI 3.0-18.2) among those insured, 5.3% points higher (95% CI 0.4-10.2) among those satisfied with their home internet connection, and 10% points higher (95% CI 4.4-16.0) among those with at least 1 medical condition. The likelihood of being offered access to a portal was 10.8% points lower (95% CI 6.3-15.2) among males and 6.9% points lower (95% CI 1.7-12.1) among those of Hispanic ethnic background.

**Table 4:** Univariable and multivariable factors associated with offering portal access.

Characteristics				Univariable analysis average marginal effect (95% CI)		*P* value		Multivariable analysis average marginal effect (95% CI)		*P* value
**Demographics**										
	**Age (years)**									
		18-40	Reference		—^a^		Reference		—	
		41-64	0.085 (0.026 to 0.145)		.005		0.025 (–0.034 to 0.084)		.40	
		More than 65	0.059 (0.012 to 0.106)		.01		0.012 (–0.061 to 0.085)		.74	
	**Sex**									
		Female	Reference		—		Reference		—	
		Male	–0.121 (–0.160 to –0.081)		<.001		–0.108 (–0.152 to –0.063)		<.001	
	**Race**									
		Asian	–0.095 (–0.236 to 0.045)		.18		0.045 (–0.159 to 0.068)		.42	
		Black	–0.039 (–0.092 to 0.015)		.15		0.003 (–0.061 to 0.069)		.91	
		Other	–0.147 (–0.283 to 0.012)		.03		–0.010 (–0.095 to 0.074)		.81	
		White	Reference		—		Reference		—	
	**Ethnicity**									
		Not Hispanic or Latino	Reference		—		Reference		—	
		Hispanic or Latino	–0.151 (–0.194 to –0.108)		<.001		–0.069 (–0.121 to –0.017)		.01	
	**Marital status**									
		Not married (other)	Reference		—		Reference		—	
		Married	0.157 (0.117 to 0.199)		<.001		0.086 (0.049 to 0.123)		<.001	
	**Education**									
		Below college level	Reference		—		Reference		—	
		College graduate or higher	0.172 (0.125 to 0.218)		<.001		0.071 (0.020 to 0.122)		.007	
	**Annual household income (US $)**									
		<50,000	Reference		—		Reference		—	
		≥50,000	0.183 (0.144 to 0.224)		<.001		0.072 (0.010 to 0.134)		.02	
	**Rural or urban communities**									
		Nonmetro	Reference		—		Reference		—	
		Metro	0.017 (–0.037 to 0.071)		.53		0.013 (–0.050 to 0.077)		.68	
**Social determinants of health**										
	**Health insurance**									
		Uninsured	Reference		—		Reference		—	
		Insured	0.242 (0.173 to 0.311)		<.001		0.106 (0.030 to 0.182)		.007	
	**Report of at least one health-related social need in the last 12 months** ^b^									
		Never	Reference		—		Reference		—	
		Yes, sometimes or often	–0.119 (–0.170 to –0.069)		<.001		–0.016 (–0.089 to 0.058)		.67	
	**Satisfaction with internet connection at home**									
		Not satisfied	Reference		—		Reference		—	
		Satisfied	0.073 (0.019 to 0.127)		.009		0.053 (0.004 to 0.102)		.04	
**Health characteristics and health care provider interactions**										
	**Medical conditions**									
		No medical conditions	Reference		—		—		—	
		At least one medical condition	0.101 (0.058 to 0.143)		<.001		0.010 (0.044 to 0.160)		.001	
	**General health**									
		Poor or fair	Reference		—		Reference		—	
		Good, very good, or excellent	0.090 (0.032 to 0.148)		.003		–0.005 (–0.057 to 0.048)		.86	
	**Confident filing medical forms**									
		No	Reference		—		Reference		—	
		Yes	0.141 (0.088 to 0.194)		<.001		0.042 (–0.012 to 0.098)		.12	

^a^Not applicable.

^b^Includes food insecurity, housing instability, and transportation access problem.

## Discussion

### Principal Findings

In this retrospective analysis of online portal access using a nationally representative sample in 2022 after the COVID-19 pandemic, we found that 61.3% of Americans use patient portals, a 27% increase compared with 48.1% in 2020 [7]. Further, 75.0% of patients were being offered access to portals by either health care provider or insurer, a 12% increase from 67.0% in 2020 [7], and 68.4% were encouraged to use portals by their provider. Both factors were associated with a significantly higher likelihood of patients’ portal use consistent with previous studies [5,9,15]; however, there remained significant disparity in portal use based on patients’ rural or urban location or education.

In recent years, governing bodies have sought to increase access to and use of patient portals while limiting information blocking, defined as a practice that interferes, prevents, or discourages the distribution of electronic health information (EHI) [7]. According to the recently passed 21st Century Cures Act (henceforth the Cures Act), EHI must be made immediately available to patients, including laboratory and imaging diagnostic tests. Several studies indicate that this increases patient ownership of their health by improving their knowledge and understanding of their health conditions [16-18]. This presents a need for equitable patient portal access, however there are barriers preventing the widespread use of portals among patients.

Despite the reported benefits of portal use, portals are not offered to approximately one-third of patients. Commonly cited reasons for not offering portals include potential increased clinical workload (eg, increased messaging to providers) as a result of patients accessing their test results before interpretation by their provider might be a contributing factor to providers not offering portals [16,17]. Other cited reasons include some providers believing there will be increased patient anxiety when reviewing results before talking to providers. However, a previous systematic review has shown a reduction or no change in patient anxiety following the use of patient portals [19].

While others have found decreased portal use among patients who are male or from minority racial or ethnic backgrounds [5,7], our study did not show any significant association with these specific factors in multivariable analysis adjusting for offering patient portal. This discrepancy is likely because males and Hispanic people were 10.8% points and 6.9% points less likely to be offered portal access. Although 79.9% of individuals of White background were offered portal access-a disproportionately higher rate than 73.8% of patients of Black background-we did not observe any significant racial difference in offering access to portals in multivariable analyses [5]. Interestingly, one study reported no significant differences in understanding of EHI among users regardless of race, suggesting that equitable access opportunities can increase portal use [5]. Our study underscores provider education to reduce bias when offering portals.

Furthermore, our study showed that college graduation and living in urban regions were independently associated with increased patient portal use, consistent with previous literature [20,21], and lower portal use among those with lower levels of education or living in rural areas will not improve with increased offering of portal. For these populations, access or digital literacy barriers likely contribute to lower portal use. Tailored interventions to increase digital health literacy can further facilitate portal use [8,22].

In our study, 43.7% (82,620,907/188,860,031) of patients reported using multiple patient portals. An additional potential barrier to access is the overwhelming number of patient portals that exist, which are rarely integrated with one another, potentially hindering motivation to use portals. This might be seen more pronounced among patients with multiple health conditions, which may require them to use different patient portals for different providers they visit [23]. Though developing more integrated patient portals would be ideal, factors such as stakeholder alignment and differential IT infrastructure among hospitals make this a difficult feat to attain [24].

As we strive to increase equitable portal access, an expansion in patient portal function may also become more prominent. Currently, patients can communicate with providers between appointments and view a variety of diagnostic tests and imaging results, however, the potential of the tool as a self-management health aid is being explored within the literature. For example, a self-assessment survey was tested among patients with chronic kidney disease in outpatient clinics [25]. This offers patients access to printouts containing learning needs and treatment plans as well as the opportunity to create physician-accessible notes allowing their providers to relay take-home messages for individual patients. Another portal centered around diabetes mellitus care called Diabetes NetPLAY fostered weekly communication for weight management through social media-type posts containing a weekly topic, research, fitness tips, and physical activity myths, as well as interactive components such as physical activity logbooks and weekly counselor messages [25]. Another study suggested that portals can be used to identify lung cancer screening candidates through an algorithm considering patient age and smoking history [26]. This population-level intervention through portals can facilitate earlier detection of malignancy, leading to better patient prognoses and lower mortality. While these functionalities may be forecasted to improve health outcomes, it is important to emphasize the need for increased access to patient portals as these technologies will only benefit patients who use the portals to their full capacity. Creating flyers about portal access and posting them in the waiting area and exam rooms, use of printed after-visit summaries with instructions on how to use portals, and sending patients an enrollment invitation to access their results after a visit are some of the ways to increase portal offerings and encouragement among patients. A systematic review has shown interventions focusing on technical training and assistance for patients may increase portal use in vulnerable populations [27].

### Strengths and Limitations

Notable strengths of this study include the use of a large database with of 5958 patients and an ethnically diverse patient population. A major limitation of this study is the depth through which questions were answered by participants. For example, patients were never prompted to discuss specific reasons as to why they chose to use or not use portals. This makes it difficult to discern what patients may subjectively feel is their largest obstacle or barrier to using their online health portals. Future studies assessing factors associated with portal use ought to better determine the exact reasons why patients may not use portals. In doing so, these answers can then be correlated with demographic factors, as done by our study, to elicit a better understanding of the obstacles to portal use amongst larger patient populations.

### Conclusion

The findings of this cross-sectional analysis of the 2022 HINTS 6 database identified several factors associated with increased portal use, such as college education, living in urban regions, access to portals offered by health care providers or insurers, and encouraging use of portals. We further identified disparities in offering of portals to patients, with married individuals, college graduates, those with an annual household income of US $50,000 or more, insured, satisfied with their home internet connection, and those with at least one medical condition being more likely to be offered portal access, while men and those of Hispanic ethnic background being less likely. The results of this study highlight the opportunity to advance equitable portal access and patient empowerment through physician encouragement of patient portal use.

## Abbreviations

EHI: electronic health information
HINTS 6: Health Information National Trends Survey

## Data Availability

The HINTS 6 database is publicly available for download. Furthermore, the recoded datasets generated and analyzed during this study are available from the corresponding author upon request.
